# Mitochondria-Associated mRNAs Restore ATP During Oxidative Stress via Cytosolic Translation

**DOI:** 10.3390/antiox15050580

**Published:** 2026-05-03

**Authors:** Dong-Bin Back, Gen Hamanaka, Ji-Hyun Park, Shin Ishikane, Masayoshi Tanaka, Takafumi Nakano, Yoshihiko Nakamura, Kazuhide Hayakawa

**Affiliations:** 1 Neuroprotection Research Laboratories, Departments of Radiology and Neurology, Massachusetts General Hospital and Harvard Medical School, Charlestown, MA 02129, USA; dback3@mgh.harvard.edu (D.-B.B.); ghamanaka@mgh.harvard.edu (G.H.); jpark79@mgh.harvard.edu (J.-H.P.); ishikane@med.uoeh-u.ac.jp (S.I.); masayoshitnk-res@outlook.jp (M.T.); naka0625@fukuoka-u.ac.jp (T.N.); nakamura58@adm.fukuoka-u.ac.jp (Y.N.); 2 Department of Pharmacology, School of Medicine, University of Occupational and Environmental Health, Fukuoka 807-8555, Japan; 3 Department of Clinical Pharmacy, Oita University Hospital, Hasama-machi, Oita 879-5593, Japan; 4 Department of Physiology and Pharmacology, Faculty of Pharmaceutical Sciences, Fukuoka University, Fukuoka 814-0180, Japan; 5 Department of Emergency and Critical Care Medicine, Fukuoka University Hospital, Fukuoka 814-0133, Japan

**Keywords:** mitochondria, MitoCoat, lipid modification, mRNA

## Abstract

Mitochondrial transplantation has been proposed as a strategy to restore cellular bioenergetics after oxidative injury, but the mechanisms governing ATP recovery remain unclear. Using placental mitochondria, we examined ATP restoration following H_2_O_2_-induced oxidative stress. Unmodified mitochondria modestly increased ATP under baseline conditions but failed to restore ATP after injury. In contrast, lipid-coated mitochondria (MitoCoat) and lipid-encapsulated mitochondria-associated mRNAs (MitoCoat–mRNA) significantly increased ATP levels in injured cells. Transcriptomic analyses revealed that ATP recovery occurred without the normalization of canonical glycolytic or oxidative phosphorylation (OXPHOS) gene programs. Instead, unmodified mitochondria induced broad transcriptional responses associated with immune activation and cellular stress, whereas MitoCoat elicited a more restricted transcriptional profile. Notably, mitochondria-associated mRNAs alone restored ATP without detectable changes in host transcriptional programs. The removal of mitochondrial surface-associated ribosomes or the inhibition of cytosolic but not mitochondrial translation attenuated ATP recovery. The restoration of key metabolic enzymes through cytosolic translation, including PFKP, pyruvate dehydrogenase, and ATP synthase subunit ATP5A suggests that mitochondria-associated mRNAs promote recovery by re-establishing coupling between glycolysis and mitochondrial OXPHOS. Together, these findings identify encapsulated mitochondria-associated mRNAs as a potential strategy to restore cellular bioenergetics under oxidative stress.

## 1. Introduction

Mitochondria are essential metabolic organelles that sustain cellular viability through ATP production, fatty-acid oxidation, and calcium buffering [1,2,3,4]. In the central nervous system (CNS), mitochondrial dysfunction is tightly linked to oxidative stress, inflammasome activation, and mitochondrial membrane permeability defects [5], processes that amplify neuronal injury and neuroinflammation across diverse conditions, including stroke, traumatic brain injury, and neurodegenerative disease [6,7]. Consequently, restoring mitochondrial function remains a major objective in efforts to promote neural resilience and repair.

Beyond their intracellular roles, mitochondria also participate in non-cell-autonomous signaling. Extracellular mitochondria have been detected in the brain and cerebrospinal fluid, and intercellular mitochondrial transfer between glia and neurons is now recognized as a mechanism supporting metabolic stability after injury [8,9]. These observations have spurred interest in mitochondrial transplantation as a therapeutic strategy for neurological injury. However, despite growing experimental and translational efforts, the specific mitochondrial components responsible for cytoprotection and metabolic recovery remain poorly defined.

Recent work has revealed that cytosolic ribosomes localize to the outer mitochondrial membrane [10,11], raising the possibility that mRNAs may associate with mitochondria as part of a spatially organized translational system. Supporting this concept, the delivery of mitochondria carrying mRNAs or mRNAs alone into macrophages enhances phagocytic activity [12], consistent with previous reports demonstrating that mitochondrial transfer promotes macrophage phagocytosis through the activation of oxidative phosphorylation (OXPHOS) [13]. These observations suggest that mitochondria-associated mRNAs can be functionally translated in recipient cells and may represent an exploitable mechanism for supporting intracellular metabolic homeostasis. Whether such mitochondria-associated mRNAs contribute directly to ATP homeostasis during oxidative stress, however, remains unknown.

We previously demonstrated that coating mitochondria with artificial DOTAP/DOPE lipid membranes enhances mitochondrial delivery and reduces cellular stress responses compared with unmodified mitochondria [14]. Here, we extend this work by isolating mitochondria-associated mRNAs and delivering them independently using lipid encapsulation. By directly comparing unmodified mitochondria, lipid-coated mitochondria (MitoCoat), and lipid-encapsulated mitochondria-associated mRNAs (MitoCoat–mRNA), we examine whether mRNA delivery alone is sufficient to restore ATP production during oxidative stress. Using combined ATP, transcriptomic, and loss-of-function analyses, we identify a translation-dependent mechanism of metabolic rescue that operates independently of nuclear gene reprogramming, revealing a previously unrecognized role for mitochondria-associated mRNAs in maintaining cellular energy homeostasis.

## 2. Materials and Methods

### 2.1. Animals

All experiments were performed following Institutional Animal Care and Use Committee protocol (Massachusetts General Hospital, 2010N000228, 4 August 2016) in accordance with the National Institutes of Health guidelines and with the United States Public Health Service’s Policy on Human Care and Use of Laboratory Animals and followed Animals in Research: Reporting In Vivo Experiments (ARRIVE) guidelines. All pregnant rats were (up to one per cage) maintained in a controlled pathogen-free/germ-free environment with a temperature of 68–73 °F, 12/12 h light/dark cycle, 30–70% humidity, and with food (Prolab Isopro RMH3000 Irradiated, 3003219-249) and water provided ad libitum.

### 2.2. Primary Cultures

For astrocyte isolation, cerebral cortices from 1–2 day-old Sprague Dawley rats were dissected, minced, and dissolved. Dissociated cells were plated in poly-D-lysine-coated 75-cm^2^ flasks and maintained in Dulbecco’s Modified Eagle’s medium containing 20% heat-inactivated fetal bovine serum (FBS) and 1% penicillin/streptomycin. After the cells were confluent (~10 days), the flasks were shaken for 1 h on an orbital shaker (220 rpm) at 37 °C to remove microglia. After shaking the flasks for 1 hour, the medium was changed with a new medium and shaken overnight (~20 h). The cells were then collected and plated for culturing astrocytes. Bone marrow-derived mesenchymal stem cells (BM-MSCs) and adipose-derived stem cells (ASCs) were isolated from 8-week-old Sprague Dawley rats. For BM-MSC isolation, femurs were dissected and cleaned of surrounding muscles and connective tissues, and the bone marrow was flushed with approximately 20 mL of PBS using a sterile syringe fitted with a 16-gauge needle after opening both ends of the bone, allowing the marrow to be expelled from the femoral cavity. The suspension was centrifuged at 1000 rpm for 3–5 min at room temperature, and the resulting cell pellets were resuspended in α-Minimum Essential Medium with nucleosides (α-MEM) containing 10% FBS and 1% penicillin for expansion of adherent cells. For ASC isolation, visceral adipose tissue was minced and dissolved with collagenase (Sigma-Aldrich, St. Louis, MO, USA, C2674) at 2 µg/mL in PBS at 37 °C with shaking at 140 rpm for 30 min. The digested suspension was filtered through a 100 µm sterile cell strainer to remove undissolved tissue fragments, followed by centrifugation at 1000 rpm for 3–5 min at room temperature. The resulting cell pellets were resuspended in the same culture medium used for BM-MSCs. All primary cell cultures used in this study were routinely characterized for purity using established lineage-specific markers. Astrocyte cultures contained >90% GFAP-positive cells, as assessed by immunostaining and adipose-derived stem cells (ASCs) and bone marrow-derived mesenchymal stem cells (BM-MSCs), and showed >95% positivity for mesenchymal markers CD29 and CD90 by flow cytometry analysis in our laboratory [9,15], confirming the high purity of the primary cell populations used in our experiments.

### 2.3. Cell Preparation and Treatments

Primary astrocytes, bone marrow-derived mesenchymal stem cells (BM-MSCs), and adipose-derived stem cells (ASCs) were seeded 3–4 days prior to experimental treatments to reach approximately 70–80% confluence at the time of treatment. Cells were plated in 96-well plates (100 µL culture medium per well) for ATP analysis, 12-well plates (1 mL medium per well) for inhibitor treatment and immunofluorescence analysis, and 6-well plates (2 mL medium per well) for Western blotting and RNA sequencing experiments. Seeding densities were determined using the 96-well plate as the reference condition, with adjustments based on differences in proliferation rate, growth characteristics, and doubling time among the cell types. Astrocytes were seeded at 7000 cells per well, whereas BM-MSCs and ASCs were seeded at 4000 cells per well in 96-well plates. For larger plate formats, the number of seeded cells was scaled proportionally according to the well surface area to maintain comparable cell density across different culture plates.

H_2_O_2_ treatment: Hydrogen peroxide (H_2_O_2_; Sigma-Aldrich, H3410, 30 wt.% in H_2_O) was used to induce oxidative stress. A 10 mM intermediate stock was freshly prepared in sterile distilled water immediately before use. Cells were treated with H_2_O_2_ at a final concentration of 100 µM for 30 min at 37 °C in a humidified 5% CO_2_ incubator by adding plate-specific volumes of the 10 mM H_2_O_2_ stock (1 µL, 10 µL, and 20 µL to 96-well, 12-well, and 6-well plates, respectively), while vehicle control wells received the same volume of sterile distilled water. After treatment, cells were washed twice with PBS and the medium was replaced with fresh culture medium. Cells were subsequently treated with exogenous mitochondria-enriched fractions, MitoCoat, or MitoCoat–mRNA. Mitochondria or lipid-coated mitochondria (MitoCoat) were applied to cultured cells at 5 or 10 µg per 100 µL medium in 96-well plates, and MitoCoat–mRNA was applied at 100 ng per 100 µL medium. For other plate formats, the amounts of mitochondria and mitochondrial RNA were scaled proportionally according to culture volume to maintain equivalent final concentrations. MitoCoat and MitoCoat–mRNA were prepared using a dual-tube lipid encapsulation system for mitochondria-enriched fractions and mitochondria-associated mRNA delivery, as previously reported by our group [16]. Following treatment, cells were analyzed using assay-specific endpoints, including ATP measurement, fluorescence imaging, Western blotting, or RNA sequencing, depending on the experimental objective.

Inhibitors and rSQSTM1 treatment: Chlorpromazine hydrochloride (CPZ; Sigma-Aldrich, C8138), cycloheximide (CHX; Sigma-Aldrich, C1988), and chloramphenicol (CAP; Sigma-Aldrich, C0378) were used to inhibit specific cellular pathways. CPZ inhibits clathrin-mediated endocytosis, CHX inhibits cytosolic translation, and CAP inhibits mitochondrial translation. Stock solutions were prepared by dissolving CPZ in sterile distilled water (5 mg/mL), CHX in dimethyl sulfoxide (DMSO; 5 mg/mL), and CAP in ethanol (1 mg/mL), and then were stored at −20 °C. For inhibitor treatment, stock solutions were diluted directly into the culture medium to achieve final concentrations of 50 µg/mL for CPZ and CHX and 10 µg/mL for CAP, and vehicle control wells received the corresponding solvent at the same volume. In treatment experiments, the inhibitors CPZ, CHX, or CAP were added simultaneously with exogenous unmodified mitochondria, MitoCoat, or MitoCoat–mRNA and maintained in the culture medium for 3 h. For fluorescence imaging experiments assessing mitochondrial uptake, exogenous unmodified mitochondria and CPZ-treated MitoCoat were labeled with MitoTracker Red CMXRos (Thermo Fisher Scientific, Waltham, MA, USA, M7512) at 100 nM in suspension prior to treatment. To examine the enhancement of unmodified mitochondria uptake by recombinant SQSTM1 (rSQSTM1; Novus Biologicals, NBP1-44490, 0.5 mg/mL stock), rSQSTM1 was added to the culture medium at a final concentration of 1 µg/mL during exposure to unmodified mitochondria. To assess CPZ-mediated inhibition of MitoCoat uptake, cells were treated with MitoCoat co-treated with or without CPZ and imaged at the end of the treatment period. For experiments examining the effects of CHX or CAP on MitoCoat–mRNA-mediated responses, cells were washed twice with PBS and collected for Western blotting on the following day.

### 2.4. Mitochondrial Isolation

Mitochondria-enriched fractions were isolated from the cryopreserved placenta, liver, spleen, heart, lung, kidney, brain, and skeletal muscle of Sprague Dawley rats using a stepwise centrifugation method, ensuring that all procedures were conducted at 4 °C to maintain mitochondrial integrity. First, the mitochondrial isolation buffer was prepared, consisting of 10 mM HEPES (pH 7.5), 250 mM sucrose, 1 mM ATP, 0.1 mM ADP, 5 mM sodium succinate, and 2 mM K_2_HPO_4_. Next, tissue samples were thawed on ice and rinsed with ice-cold mitochondrial isolation buffer to remove blood and contaminants. Then, the tissue was transferred to a pre-chilled glass tissue grinder containing 1 mL of mitochondrial isolation buffer. Afterward, homogenization was performed using 20–30 strokes of a tight-fitting glass rod to ensure thorough mechanical disruption while minimizing mitochondrial damage. The homogenate was subsequently subjected to a series of centrifugation steps. Initially, centrifugation at 1000× *g* for 5 min at 4 °C was performed to remove cellular debris and nuclei. The supernatant was then centrifuged at 4000× *g* for 5 min at 4 °C to remove some larger organelles, such as fragments of the Golgi apparatus and partially sedimented lysosomes. The resulting supernatant was then centrifuged at 8000× *g* for 10 min at 4 °C, yielding a mitochondrial-enriched pellet. The final pellet was resuspended in PBS to achieve a protein concentration of 1 μg/μL—as determined by a Bradford protein assay—using gentle pipetting to ensure uniform suspension, and then stored on ice for immediate use.

### 2.5. Flow Cytometry (FC) Analysis

Standard FC analysis was performed with BD Fortessa 20. Placental mitochondria were stained with Mitotracker DR (200 nM, Thermo Fisher Scientific, M22426) or Calnexin antibody (5 μg/mL, Novus Biologicals, NB100-1965B). FC analysis was performed using a variety of controls, including unstained samples and single-stained samples for determining appropriate gates, voltages, and compensations required in multivariate flow cytometry.

### 2.6. Trypsin Treatment of Isolated Mitochondria

Isolated mitochondria-enriched fractions resuspended in mitobuffer were incubated with trypsin–EDTA (0.25%, Gibco, 25200056) at a 1:1 volume ratio for 30 min at 37 °C. The reaction was neutralized with cell culture medium at a trypsin-to-medium ratio of 1:3, followed by two PBS washes. After each wash, mitochondria were pelleted at 8000× *g* for 10 min. The final pellet was resuspended in mitobuffer and used for downstream Western blotting and immunofluorescence analyses. Control mitochondria were processed in parallel under identical conditions without trypsin treatment.

### 2.7. mRNA Isolation

Total RNA, including mitochondrial mRNA, was extracted from mitochondria-enriched fractions using the RNeasy Micro Kit (QIAGEN) according to the manufacturer’s protocol. After mitochondrial isolation, the mitochondrial pellet was resuspended in 350 μL of RLT buffer, supplied with the kit, and supplemented with 1% β-mercaptoethanol to ensure the complete lysis of mitochondria and inactivation of RNases. The sample was then homogenized by pipetting up and down 10 times, followed by vortexing for 30 s to enhance mitochondrial membrane disruption. To facilitate RNA binding to the silica membrane in the RNeasy spin column, the lysate was mixed with an equal volume of 70% ethanol (350 μL). The mixture was then transferred to the spin column and centrifuged at 8000× *g* for 15 s at room temperature, allowing the RNA to bind efficiently to the membrane. To remove contaminants such as proteins and residual DNA, the column was first washed with 500 μL of RW1 buffer. Subsequently, on-column DNase I treatment was performed by adding 80 μL of DNase incubation buffer, followed by incubation at room temperature for 15 min to eliminate genomic DNA contamination. The column was then washed twice with 500 μL of RPE buffer to further improve RNA purity. The purified RNA was eluted using 14 μL of RNase-free water and immediately stored at −80 °C for further analysis.

### 2.8. Coating with DOTAP/DOPE

Add 300 µL of mineral oil to a 1.5 mL tube, then supplement it with 3 µL of 100 mM DOPE (prepared by dissolving 100 mg DOPE in 200 µL chloroform and 1140 µL ethyl alcohol) and 1 µL of 300 mM DOTAP (prepared by dissolving 50 mg DOTAP in 100 µL chloroform and 140 µL ethyl alcohol). Mix thoroughly and transfer 200 µL of this lipid-containing mineral oil into a new tube. Combine the mitochondrial suspension (up to 100 µg protein or the equivalent mRNAs in 10 µL containing 1% polyvinyl alcohol) with the 200 µL lipid-containing mineral oil and generate a water-in-oil (W/O) emulsion by gently pipetting 10–15 times. In parallel, prepare a second tube containing 500 µL of PBS overlaid with 100 µL of mineral oil supplemented with 1 mM DOPE and DOTAP. Carefully layer the W/O emulsion onto the prepared oil–PBS interface and allow it to stand for 5 min after slow addition. The sample can then be centrifuged at 12,000× *g* for 10 min at 4 °C. Following centrifugation, discard the supernatant and gently resuspend the pellet in an appropriate buffer before use [16].

### 2.9. ATP Measurement

ATP was determined with CellTiter-Glo luminescence (Promega). Standard ATP was used for the measurement of ATP content (% of control) in cells. ATP values were normalized to the corresponding uninjured control group within each experiment.

### 2.10. Cell Number/Survival Assay

Cell proliferation/survival was assessed using a WST reduction assay (Dojindo), which detects the dehydrogenase activity of viable cells. The cells were incubated with 10% WST solution for 1 h at 37 °C. Then, the absorbance of the culture medium was measured with a microplate reader at a test wavelength of 450 nm and a reference wavelength of 630 nm.

### 2.11. Fluorescence Quantification

Mitochondrial uptake efficiency was evaluated by measuring the MitoTracker-positive area using Fiji (ImageJ) following the thresholding of fluorescence signals. The signal was normalized to a cell number, determined by counting DAPI-positive nuclei in each field, and expressed as mitochondrial area per cell. Values were further normalized to the mean of the corresponding control group and presented as a percentage. Colocalization between fluorescent signals was quantified using ImageJ (NIH) with the Colocalization Finder plugin. Fluorescence images were acquired under identical imaging settings and imported into ImageJ for analysis. Regions of interest were defined for each image, and consistent thresholding was applied across all samples to minimize background signal. Pearson’s correlation coefficient (Rr) was calculated to assess the degree of colocalization between channels. For each condition, multiple images were analyzed and averaged to obtain representative values.

### 2.12. Western Blot Analysis

Each sample was loaded onto 4–20% Tris-glycine gels. After electrophoresis and transferring to nitrocellulose membranes, the membranes were blocked in Tris-buffered saline containing 0.1% Tween 20 and 0.2% I-block (Tropix, T2015) for 90 min at room temperature. Membranes were then incubated overnight at 4 °C with the following primary antibodies: anti-β-actin (1:1000, Sigma-Aldrich A5441), glycolysis detection antibodies (1:500, Cell Signaling, 8377T), and total OXPHOS rodent WB antibody cocktail (1:200, Abcam, ab110413). After incubation with peroxidase-conjugated secondary antibodies, visualization was enhanced by chemiluminescence (GE Healthcare, NA931-anti-mouse, or NA934-anti-rabbit, or NA935-anti-rat). Optical density was assessed using the NIH Image analysis software.

### 2.13. Detection of Ribosome-Associated Proteins on Isolated Mitochondria

To detect ribosome-associated proteins on isolated mitochondria, mitochondria-enriched fractions were subjected to double staining with MitoTracker and a FITC-conjugated antibody against ribosomal protein S6 (phospho S235/S236) (Abcam, ab278685). Following isolation, mitochondria were resuspended in PBS, and 50 µg mitochondrial protein was used for each staining reaction performed in a final volume of 100 µL containing 2 µL FITC-conjugated anti-RPS6 antibody (final dilution 1:50) and MitoTracker (100 nM). The samples were gently mixed and incubated for 30 min at 37 °C without agitation. After staining, mitochondria were washed twice with PBS, centrifuged at 8000× *g* for 10 min, and resuspended in 100 µL PBS, and a portion of the suspension was transferred onto poly-D-lysine-coated glass surfaces for fluorescence observation.

### 2.14. Fluorescent Labeling of Mitochondria-Associated mRNA for MitoCoat–mRNA Uptake Imaging

Mitochondria-associated mRNA was fluorescently labeled using the Label IT^®^ Fluorescein Nucleic Acid Labeling Kit (Mirus Bio, MIR 3210) according to the manufacturer’s instructions, with minor modifications. Labeling reactions were prepared in sterile tubes in a final volume of 50 µL containing mitochondria-associated mRNA, 10× Labeling Buffer A (5 µL), Label IT^®^ Reagent, and RNase-free water to adjust the final volume to 50 µL. Five µL of Label IT^®^ Reagent was added to obtain a Label IT^®^ Reagent-to-RNA ratio of approximately 0.5–1.0:1.0 (µL:µg), within the range recommended in the manufacturer’s instructions. The volume of mitochondria-associated mRNA was adjusted depending on the Label IT^®^ Reagent-to-RNA ratio, the stock concentration of mitochondria-associated mRNA, the number of wells used for downstream experiments, and the final target concentration of MitoCoat–mRNA (100 ng per 100 µL, corresponding to the condition used in 96-well experiments). For fluorescence imaging experiments, the labeling reaction was incubated at 37 °C for 1 h and purified using G50 spin columns (included in the labeling kit) to remove the unreacted labeling reagent. Prior to sample loading, the columns were centrifuged at 735× *g* for 1 min twice to remove the storage buffer. The 50 µL labeling reaction was then applied to the column and centrifuged at 735× *g* for 2 min to collect the purified fluorescently labeled mitochondria-associated mRNA. The purified labeled RNA was used for MitoCoat–mRNA preparation using the dual-tube lipid encapsulation system, as previously reported by our group [16]. Astrocytes were stained with MitoTracker (100 nM, 30 min at 37 °C) to label astrocytic mitochondria and then washed with PBS. Cells were then treated with fluorescently labeled MitoCoat–mRNA for 1 h, and fluorescence images were acquired after incubation without additional washing steps.

### 2.15. RNA Profiling

Three RNA samples from each experimental group were randomly selected for bulk RNA sequencing. Library preparation and sequencing were performed with GENEWIZ (USA). Raw reads were trimmed to remove adapter sequences and low-quality bases using Trimmomatic (v0.36) and aligned to the *Rattus norvegicus* reference genome (Rnor6.0, Ensembl annotation) using the splice-aware aligner STAR aligner (v2.5.2b). Alignment files (BAM) were generated for downstream analysis. Gene-level counts were quantified using featureCounts from the Subread package (v1.5.2), counting uniquely mapped reads overlapping annotated exons (strand-specific when applicable). Differential expression analysis was performed using DESeq2 with Wald tests, and genes with an adjusted *p*-value (FDR) < 0.05 were considered significantly differentially expressed. RNA-seq data have been deposited in the NCBI Gene Expression Omnibus under accession number GSE326220 and in the Sequence Read Archive under BioProject accession number PRJNA1443374.

### 2.16. Statistical Analysis

Data are presented as mean ± SD. All in vitro experiments were performed with at least duplicate samples and repeated independently at least three times. Investigators were not blinded during in vitro studies; however, the samples were randomized, and no data were excluded from the analyses. Statistical analyses were performed using GraphPad Prism version 9. Comparisons between two groups were made using an unpaired two-tailed *t*-test. Comparisons among multiple groups were performed using one-way ANOVA followed by Tukey’s multiple-comparison test. Statistical significance was defined as *p* < 0.05.

## 3. Results

### 3.1. Validation of Isolated Mitochondria in Flow Cytometry, Western Blot, and mRNA Profiling

**Figure 1:**
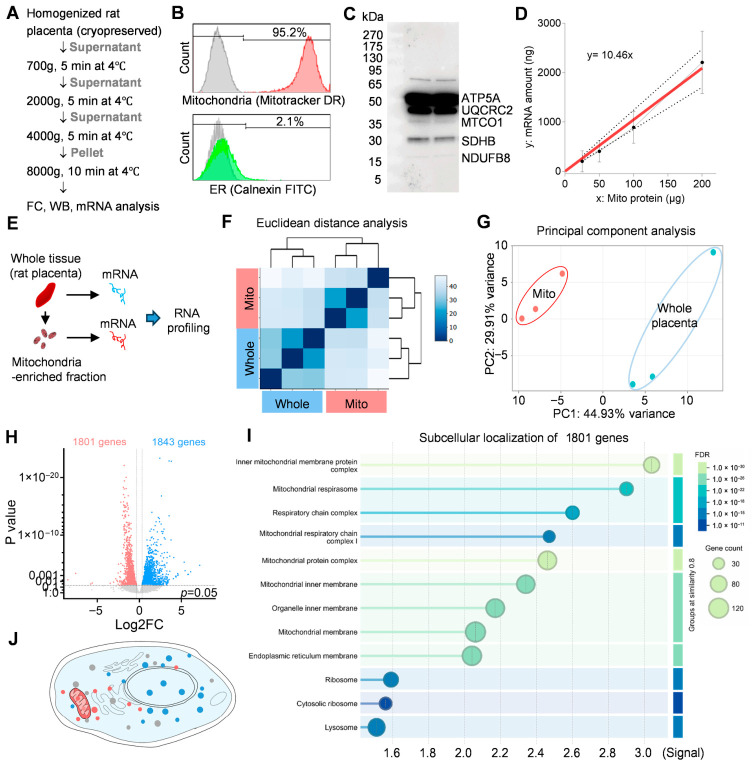
Validation of mitochondria-enriched fractions and associated mRNA profiles. (**A**) Schematic of mitochondrial isolation from cryopreserved rat placenta followed by validation using flow cytometry, Western blotting, and RNA profiling. (**B**) Flow cytometric analysis showing that >90% of isolated particles were positive for MitoTracker and fewer than 3% for ER (Calnexin), indicating high mitochondrial enrichment. Gray peak indicates negative control (unstained control). (**C**) Western blot analysis confirming robust expression of mitochondrial oxidative phosphorylation (OXPHOS) proteins in the isolated fraction. (**D**) Detection of mRNAs in mitochondria-enriched preparations, demonstrating retention of RNA species following isolation. (**E**) Experimental schematic for RNA sequencing by using isolated whole placenta tissue (n = 3) and the mitochondria-enriched fraction-derived mRNA (n = 3). (**F**,**G**) Euclidean distance analysis and principal component analysis (PCA) showing clear separation between whole placenta and mitochondria-enriched samples. (**H**) Volcano plot identifying genes significantly enriched in the mitochondrial fraction relative to whole placenta tissue. (**I**,**J**) Subcellular localization analysis of enriched transcripts, revealing predominant association with mitochondrial membranes, respiratory chain complexes, and mitochondrial protein complexes, with minor contributions from other compartments.

We first examined mitochondrial enrichment in our samples. Cryopreserved rat placenta was homogenized and subjected to sequential centrifugation, with the final pellet collected after centrifugation at 8000× *g* for 10 min at 4 °C. These samples were then analyzed using flow cytometry, Western blotting, and mRNA profiling (Figure 1A). Flow cytometry showed that more than 90% of particles were MitoTracker-positive, whereas fewer than 3% were identified as ER-derived components (Figure 1B), and Western blotting confirmed the robust expression of mitochondrial OXPHOS proteins (Figure 1C). Using these mitochondria-enriched samples, we consistently detected mRNAs (Figure 1D).

We next performed RNA profiling on whole placenta and the mitochondria-enriched fraction (Figure 1E). Euclidean distance analysis and principal component analysis demonstrated that the mitochondria-enriched fraction displayed a distinct gene expression profile compared with whole placenta tissue (Figure 1E–G). A volcano plot identified 1801 genes significantly enriched in the mitochondrial fraction (Figure 1H). Subcellular localization analysis further showed that these genes were predominantly associated with the mitochondrial membrane, respiratory chain complexes, and mitochondrial protein complex, although a small subset related to the endoplasmic reticulum, cytosolic ribosomes, and lysosomes was also detected (Figure 1I,J).

### 3.2. Lipid-Coated Mitochondria or Mitochondria-Associated mRNAs Restore ATP Levels During Oxidative Stress

**Figure 2:**
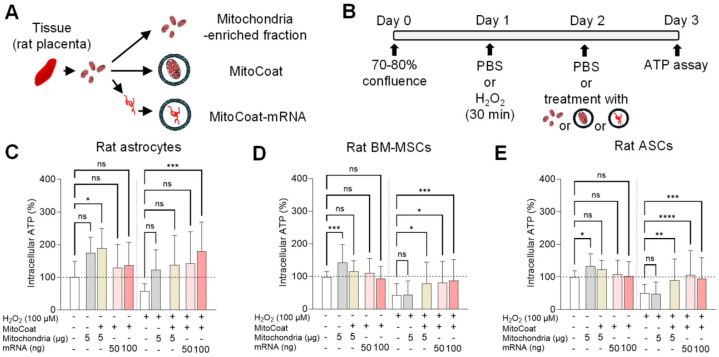
Lipid-coated mitochondria and mitochondria-associated mRNAs restore ATP levels during oxidative stress. (**A**) Experimental schematic illustrating preparation of unmodified mitochondria, lipid-coated mitochondria (MitoCoat), and lipid-encapsulated mitochondria-associated mRNAs (MitoCoat–mRNA). (**B**) Experimental design for ATP measurement in rat cortical astrocytes, bone marrow-derived mesenchymal stem cells (BM-MSCs), and adipose-derived stem cells (ASCs) subjected to H_2_O_2_-induced oxidative stress. (**C**–**E**) Quantification of intracellular ATP levels under baseline and oxidative stress conditions following treatment with unmodified mitochondria, MitoCoat, or MitoCoat–mRNA. Under oxidative stress, MitoCoat and MitoCoat–mRNA significantly restored ATP levels, whereas unmodified mitochondria did not. n = 12–14 in astrocytes, n = 41–55 in BM-MSCs, and n = 42–49 in ASCs from three to six independent experiments; * *p* < 0.05, ** *p* < 0.01, *** *p* < 0.001, **** *p* < 0.0001, one-way ANOVA followed by Tukey’s test. Data are shown as mean ± SD.

Next, the isolated mitochondria from rat placenta were divided into three groups: unmodified mitochondria, MitoCoat, and MitoCoat-mRNA that were extracted and subsequently encapsulated in DOTAP/DOPE lipids (Figure 2A). Our strategy of coating mitochondria with artificial cationic and fusogenic lipids was designed to prevent unwanted toxicity via damaged mitochondria, enhance mitochondrial delivery, and improve cerebroprotective efficacy [16]. To evaluate cellular responses in both the CNS and peripheral compartments, we applied these mitochondria-based treatments to rat cortical astrocytes, BM-MSCs, and ASCs exposed to H_2_O_2_-induced oxidative stress and evaluated their effects on ATP restoration (Figure 2B). These cell types were selected because astrocytes are central mediators of metabolic and oxidative stress responses in the injured CNS and can transfer mitochondria to neurovascular cells [9,17,18], whereas BM-MSCs and ASCs represent clinically relevant regenerative cell populations known to participate in mitochondrial transfer and metabolic rescue in peripheral organs such as the heart and lungs [19,20]. Together, these features suggest that enhancing mitochondrial rescue in these cell types may provide a strategy to amplify endogenous healthy mitochondrial transfer.

Under baseline conditions, unmodified mitochondria significantly increased ATP levels in BM-MSCs and ASCs and modestly elevated ATP in astrocytes, whereas MitoCoat mRNA alone had no measurable effect in any cell type (Figure 2C–E). In contrast, under oxidative stress, both MitoCoat and MitoCoat mRNA significantly restored ATP levels in injured BM-MSCs and ASCs. In astrocytes, only MitoCoat–mRNA (100 ng) produced both a robust increase in ATP levels and significantly improved cell viability (Figure 2C, Supplementary Figure S1). In contrast, MitoCoat treatment resulted in only modest increases in ATP that did not reach statistical significance, despite a significant improvement in cell viability after injury (Supplementary Figure S1) compared to untreated controls. These findings suggest a cell-type-specific response, with astrocytes exhibiting more limited changes in ATP dynamics but a potentially protective effect under oxidative stress.

### 3.3. ATP Dynamics in H_2_O_2_-Injured Astrocytes Occur Without Detectable Transcriptional Metabolic Rewiring

**Figure 3:**
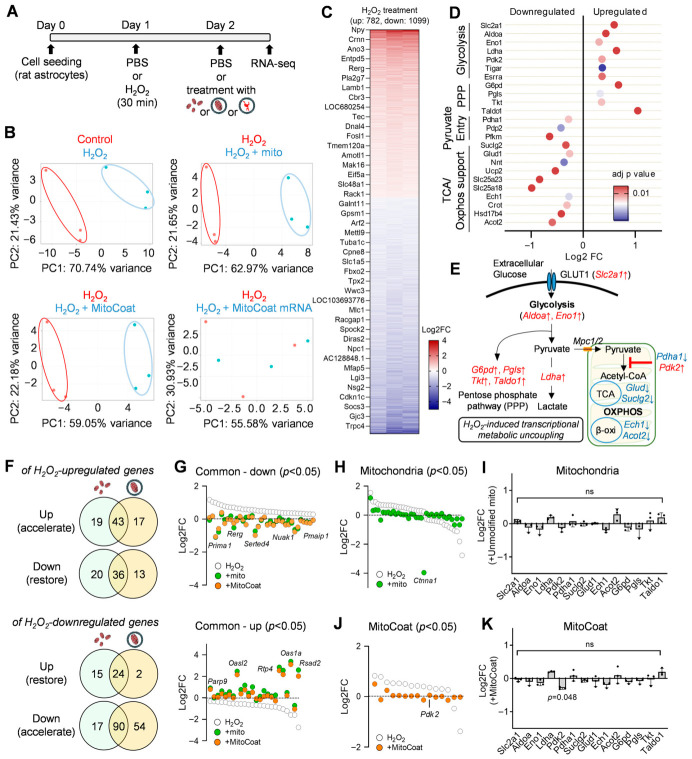
Distinct transcriptional responses to mitochondrial interventions in oxidatively injured astrocytes. (**A**) Experimental design. Astrocytes were exposed to H_2_O_2_ for 30 min, followed by treatment 24 h later with unmodified mitochondria, MitoCoat, or MitoCoat–mRNA. RNA sequencing was performed 6 h post-treatment. (**B**) Principal component analysis (PCA) showing that H_2_O_2_ markedly altered global gene expression. Both unmodified mitochondria and MitoCoat induced further transcriptional divergence from the H_2_O_2_-injured state, whereas MitoCoat–mRNA produced no detectable global transcriptional shift. (**C**) Differential gene expression analysis demonstrating that H_2_O_2_ upregulated 782 genes and downregulated 1099 genes relative to untreated controls. (**D**) H_2_O_2_-induced alteration of metabolic gene expression. Oxidative injury increased glycolytic regulatory genes while suppressing oxidative phosphorylation components. Metabolic switch genes were altered, including reduced *Pdha* and increased *Pdk2* expression, consistent with diminished pyruvate entry into mitochondria. (**E**) Schematic model illustrating injury-induced uncoupling of glycolysis from mitochondrial oxidative phosphorylation, a state predicted to limit efficient ATP production. (**F**) Quantification of partially rescued genes following treatment. Unmodified mitochondria downregulated 56 and upregulated 39 H_2_O_2_-altered genes, whereas MitoCoat downregulated 49 and upregulated 26 genes. (**G**) Shared rescue genes between unmodified mitochondria and MitoCoat were enriched for cytoskeletal, membrane, and signaling scaffold components (e.g., *Arhgap21*, *Arhgef19*, *Kif26b*, *Limk2*, *Myo1e*, *Akap13*, *Epha2*, *Papss2*, *Pmepa1*, *Phldb1*), rather than core metabolic enzymes. (**H**,**I**) Genes uniquely restored by unmodified mitochondria were associated with metabolic adaptation and stress responses (*Nrg1*, *Gdf11*, *Hspb1*, *Mgll*, *Thrsp*, *Medag*, *Slc14a1*), without normalization of ATP-relevant metabolic reprogramming. (**J**,**K**) MitoCoat reduced the expression of *Pdk2*, a negative regulator of PDH, consistent with potential facilitation of pyruvate entry into the TCA cycle. However, broader ATP-linked metabolic gene programs remained largely unchanged, indicating that ATP recovery is unlikely to result from widespread transcriptional normalization of bioenergetic pathways.

Because astrocytic mitochondrial function plays a critical role in the metabolic support and recovery of the injured CNS [9], astrocytes were used to evaluate whether mitochondrial interventions promote transcriptional normalization of bioenergetic programs under oxidative stress. To address this, we performed RNA profiling in H_2_O_2_-treated astrocytes supplemented with unmodified mitochondria, MitoCoat, or MitoCoat–mRNA. Astrocytes were first exposed to H_2_O_2_ for 30 min to induce oxidative stress and were subsequently treated 24 h later. RNA sequencing was performed 6 h after mitochondrial interventions (Figure 3A). Principal component analysis revealed that H_2_O_2_ profoundly altered global gene expression, and both isolated mitochondria and MitoCoat induced additional transcriptional divergence from the H_2_O_2_-injured state (Figure 3B). In contrast, MitoCoat–mRNA produced no detectable transcriptional changes, indicating that neither the mRNA payload nor the DOTAP/DOPE lipid coating alone drives global transcriptional reprogramming (Figure 3B). Relative to the untreated controls, H_2_O_2_ upregulated 782 genes and downregulated 1099 genes (Figure 3C). Notably, oxidative injury enhanced genes involved in the glycolytic and pentose phosphate pathway while suppressing mitochondrial pyruvate oxidation, evidenced by reduced *Pdha* and increased *Pdk2* expression, which inhibits the pyruvate dehydrogenase complex and restricts pyruvate entry into the TCA cycle (Figure 3D). In parallel, several genes supporting the TCA cycle and OXPHOS were downregulated (Figure 3D). Together, these metabolic signatures suggest that oxidative stress uncouples glycolytic metabolism from mitochondrial OXPHOS, leading to constrained ATP production (Figure 3E).

We next examined how mitochondrial interventions influenced the metabolic signatures induced by H_2_O_2_. Among the H_2_O_2_-responsive genes, unmodified mitochondria or MitoCoat treatments partially restored the transcriptional profile by downregulating 56 and 49 H_2_O_2_-induced genes, respectively, and by upregulating 39 and 26 genes suppressed by H_2_O_2_ (Figure 3F). Shared rescue signatures between unmodified mitochondria and MitoCoat were dominated by cytoskeletal and membrane or signaling scaffold genes (e.g., *Arhgap21*, *Arhgef19*, *Kif26b*, *Limk2*, *Myo1e*, *Akap13*, *Epha2*, *Papss2*, *Pmepa1*, *Phldb1*; Figure 3G), rather than core metabolic enzymes. Unmodified mitochondria restored the expression of genes involved in lipid handling, cellular homeostasis, and trophic signaling (*Nrg1*, *Gdf11*, *Hspb1*, *Mgll*, *Thrsp*, *Medag*, and *Slc14a1*), but not ATP-producing metabolic pathways (Figure 3H,I). MitoCoat normalized *Pdk2* (Figure 3J,K), thereby relieving the inhibition of pyruvate entry into the TCA cycle and supporting mitochondrial ATP production [21]. However, the expression of other ATP-linked metabolic genes remained largely unchanged (Figure 3J,K), indicating that ATP recovery following mitochondrial treatments is unlikely to arise from the widespread transcriptional normalization of bioenergetic pathways.

### 3.4. ATP Preservation by MitoCoat Reflects Enhanced Mitochondrial Uptake and Reduced ATP Consumption During Oxidative Stress

Despite ATP elevation following MitoCoat treatment, canonical ATP production and metabolic regulatory genes were not normalized, raising the question of whether ATP recovery reflects altered mitochondrial uptake and reduced intracellular energy demand rather than transcriptional metabolic reprogramming. We therefore examined uptake and intracellular handling pathways.

**Figure 4:**
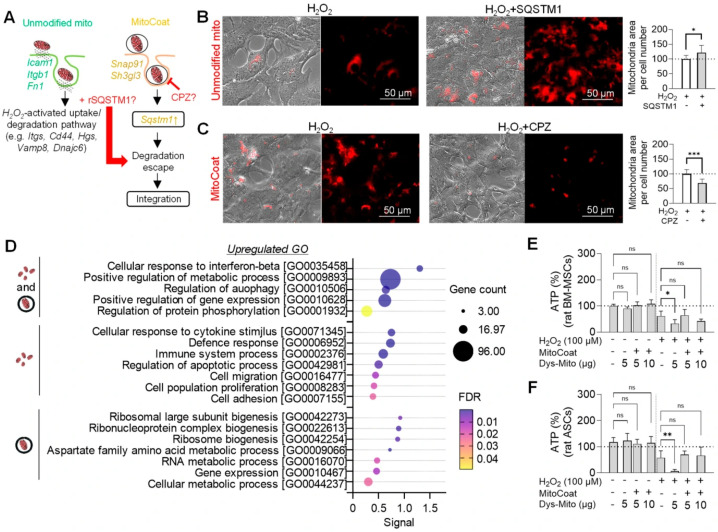
MitoCoat enhances mitochondrial uptake and reduces energetic stress in injured cells. (**A**) Model of intracellular handling following H_2_O_2_ injury. Oxidative stress induced a degradative state with increased endosomal–lysosomal components (*Hgs*, *Vamp8*, *Dnajc6*). Unmodified mitochondria preferentially upregulated adhesion-dependent uptake and endosomal routing genes (*Icam1*, *Itgb1*, *Fn1*), whereas MitoCoat enriched clathrin-associated vesicle trafficking and membrane remodeling pathways (*Snap91*, *Sh3gl3*) and uniquely induced Sqstm1, suggesting enhanced intracellular persistence. (**B**,**C**) Functional validation. SQSTM1 supplementation (1.0 μg/mL) increased intracellular MitoTracker^+^ mitochondrial signal, while inhibition of clathrin-mediated uptake with chlorpromazine (CPZ; 50 μg/mL) reduced accumulation of MitoCoat-modified mitochondria following H_2_O_2_ injury. n = 8–10 from three independent experiments; * *p* < 0.05, *** *p* < 0.001 two-sided Student *t*-test. Data are shown as mean ± SD. (**D**) Gene ontology analysis. Unmodified mitochondria induced immune activation, cytoskeletal remodeling, and stress-response pathways. In contrast, MitoCoat elicited a more restricted transcriptional profile enriched for ribosomal biogenesis, RNA metabolism, and amino acid metabolism, with minimal immune activation. (**E**,**F**) ATP measurements. Cold-stored, compromised mitochondria reduced intracellular ATP levels, whereas MitoCoat-modified mitochondria preserved ATP content under oxidative stress. (**E**) BM-MSCs, (**F**) ASCs, n = 6–7 from two independent experiment; * *p* < 0.05, ** *p* < 0.01, one-way ANOVA followed by Tukey’s test. Data are shown as mean ± SD.

H_2_O_2_ treatment induced adhesion-mediated uptake pathways, reflected by the increased expression of *Itga5* and *Cd44*, and promoted a degradative transcriptional program characterized by the upregulation of endosomal–lysosomal regulatory genes, including *Hgs, Vamp8*, and *Dnajc6*, consistent with the increased sequestration of internalized material during oxidative stress [22] in rat astrocytes. In this setting, unmodified mitochondria further upregulated adhesion and endosomal routing genes (*Icam1*, *Itgb1*, *Fn1*), suggesting enhanced integrin–ECM engagement coupled to degradative trafficking. In contrast, MitoCoat upregulated genes involved in clathrin-associated vesicle trafficking and membrane remodeling (*Snap91*, *Sh3gl3*) and uniquely induced *Sqstm1*, a mediator of lysosomal escape and enhanced mitochondrial transfer efficiency [12,23]. Based on these transcriptional differences, we hypothesized that once internalized, unmodified mitochondria are preferentially routed toward degradative compartments in oxidatively stressed astrocytes. In contrast, MitoCoat promotes clathrin-dependent internalization and facilitates SQSTM1-associated escape from degradative processing, thereby supporting intracellular persistence and potentially contributing to bioenergetic regulation (Figure 4A). Consistent with this model, SQSTM1 supplementation elevated the intracellular MitoTracker-positive mitochondrial signal of unmodified mitochondria (Figure 4B), whereas the inhibition of clathrin-mediated endocytosis with CPZ reduced the accumulation of MitoCoat-modified mitochondria following H_2_O_2_ injury (Figure 4C).

We next investigated whether differences in ATP levels were associated with changes in intracellular energy demand. Gene ontology analysis revealed that both treatments induced overlapping transcriptional programs enriched for interferon-β response, metabolic regulation, autophagy, gene expression, and protein phosphorylation (Figure 4D). Unmodified mitochondria were additionally associated with the enrichment of gene programs linked to immune activation and broader cellular responses, including the regulation of apoptosis, migration, proliferation, and adhesion (Figure 4D), processes commonly associated with cellular stress responses and increased energetic demand. In contrast, MitoCoat-treated astrocytes displayed a more restricted transcriptional profile, lacking the enrichment of cytokine or immune response pathways and instead showing the enrichment of ribosomal biogenesis, aspartate-family amino acid metabolism, and RNA metabolism (Figure 4D). Consistent with this interpretation in astrocytes, the exposure of BM-MSCs or ASCs to cold-stored, OXPHOS-compromised mitochondria further reduced intracellular ATP levels, whereas MitoCoat-modified mitochondria prevented ATP reduction (Figure 4E,F), supporting a role for MitoCoat in preserving cellular bioenergetic balance under conditions of mitochondrial stress.

Collectively, these findings support a working model in which ATP preservation by MitoCoat may arise from two convergent mechanisms: enhanced mitochondrial persistence through clathrin- and SQSTM1-associated pathways and reduced ATP expenditure due to attenuated immune and cytoskeletal responses, thereby helping maintain intracellular ATP levels.

### 3.5. Unexpected Contribution of Mitochondria-Associated mRNAs to ATP Restoration in Astrocytes

**Figure 5:**
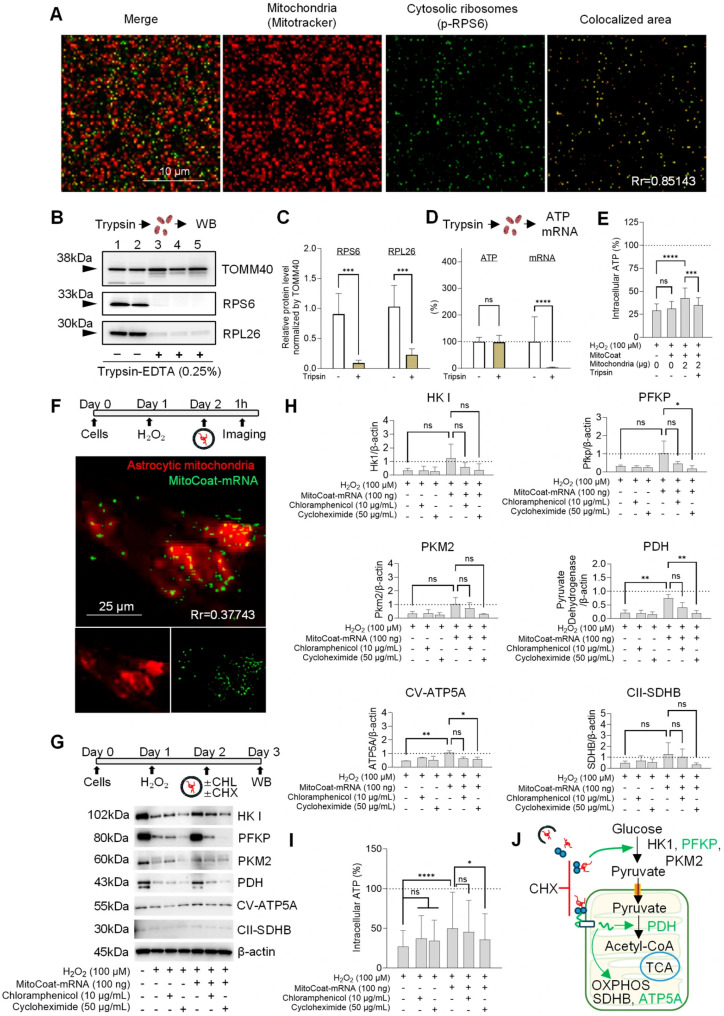
Mitochondria-associated mRNAs restore ATP through cytosolic translation in astrocytes. (**A**) Immunofluorescence showing the colocalization of MitoTracker^+^ mitochondria with the ribosomal protein RPS6, consistent with ribosome association at the outer mitochondrial membrane. Colocalization was quantified by Pearson’s correlation coefficient (Rr = 0.85143). (**B**,**C**) Western blot analysis demonstrating the presence of ribosomal proteins (RPS6, RPL26) in isolated mitochondrial fractions and their depletion following trypsin treatment. n = 5–6 from two independent experiments; *** *p* < 0.001, two-sided Student’s *t*-test. Data are shown as mean ± SD. (**D**) Quantification showing that trypsin treatment substantially reduced mitochondria-associated mRNAs without altering intrinsic mitochondrial ATP content. Although mRNA levels were markedly decreased, a small residual fraction remained detectable, likely reflecting protected or internal RNA species not accessible to proteolytic digestion. ATP: n = 24, mRNA: n = 9 from three independent experiments; **** *p* < 0.0001, two-sided Student’s *t*-test. Data are shown as mean ± SD. (**E**) ATP restoration by MitoCoat was significantly attenuated following the trypsin removal of surface-associated ribosomes and mRNAs. n = 48 from three independent experiments; *** *p* < 0.001, **** *p* < 0.0001, one-way ANOVA followed by Tukey’s test. Data are shown as mean ± SD. (**F**) Delivered mitochondria-associated mRNAs localized in proximity to mitochondria; this localization was reduced by CHX treatment. Colocalization was quantified by Pearson’s correlation coefficient (Rr = 0.37743). (**G**,**H**) Western blot analysis showing that H_2_O_2_ injury reduced glycolytic and oxidative phosphorylation proteins, while mRNA treatment partially restored these markers. ATP5A and PDH were significantly increased in a cytosolic translation-dependent manner. n = 3 from three independent experiments; * *p* < 0.05, ** *p* < 0.01, one-way ANOVA followed by Tukey’s test. Data are shown as mean ± SD. (**I**) Pharmacological inhibition of cytosolic translation with CHX (50 μg/mL) reduced ATP recovery, whereas the inhibition of mitochondrial translation with CAP (10 μg/mL) had no effect. n = 98 from seven independent experiments; * *p* < 0.05, **** *p* < 0.0001, one-way ANOVA followed by Tukey’s test. Data are shown as mean ± SD. (**J**) Working model illustrating that mitochondria-associated mRNAs support re-establishing metabolic coupling between glycolysis and mitochondrial oxidative phosphorylation and promoting ATP production in H_2_O_2_-injured cells.

In the present study, MitoCoat–mRNA restored intracellular ATP levels in H_2_O_2_-injured cells. However, the intracellular mechanisms of how the delivered mRNAs promote metabolic recovery remain unclear. To begin addressing this question, we investigated whether these effects are mediated by ribosomes and associated mRNAs localized to the mitochondrial surface. Immunofluorescence analysis demonstrated strong colocalization of MitoTracker-positive mitochondria with the ribosomal protein RPS6 (Figure 5A), consistent with previous reports [10,11].

To further examine this association, isolated mitochondria were subjected to trypsin treatment to remove outer membrane-associated translational complexes. Western blot analysis confirmed that the mitochondrial fraction contained ribosomal proteins, including RPS6 and RPL26, and that trypsin treatment markedly reduced their abundance (Figure 5B,C). Importantly, trypsin treatment did not alter ATP levels within the isolated mitochondrial fraction but substantially depleted mitochondria-associated mRNAs (Figure 5D), with a small residual fraction remaining detectable, likely reflecting protected or internal RNA species not accessible to proteolytic digestion. These findings indicate the effective removal of surface-associated cytosolic ribosomes and mRNAs while preserving intrinsic mitochondrial integrity. Consistent with this, the abundance of TOMM40 was not markedly reduced under our experimental conditions, suggesting that outer mitochondrial membrane proteins may be partially protected from proteolytic digestion or not fully accessible to trypsin. These observations indicate that trypsin treatment primarily affects surface-associated components without gross disruption of mitochondrial structure. Functional loss-of-function analyses further demonstrated that the capacity to restore ATP levels in injured cells using MitoCoat was significantly attenuated compared with trypsin-untreated MitoCoat (Figure 5E).

Next, we examined the localization of delivered mRNAs and their translation. At 1 h post-treatment, a subset of fluorescently labeled mRNAs was detected in close proximity to mitochondria (Figure 5F). Western blot analysis showed that H_2_O_2_ injury reduced the expression of proteins involved in ATP production, including glycolytic regulators (HK1, PFKP, PKM2), the pyruvate oxidation enzyme pyruvate dehydrogenase (PDH), and oxidative phosphorylation components (SDHB, ATP5A) (Figure 5G,H). mRNA treatment showed a trend toward restoring these proteins, with PDH and ATP5A significantly increasing (Figure 5G,H). Importantly, these effects were abolished by the cytosolic translation inhibitor CHX but were minimally affected by the mitochondrial translation inhibitor CAP (Figure 5G,H), indicating that the translated proteins are primarily generated through cytosolic translation.

Consistently, the pharmacological inhibition of cytosolic translation with CHX significantly reduced ATP restoration, whereas the inhibition of mitochondrial translation with CAP did not affect mRNA-mediated ATP recovery (Figure 5I). These findings suggest that the cytosolic translation of nuclear-encoded metabolic mRNAs restores key metabolic enzymes, thereby re-establishing metabolic coupling between glycolysis and mitochondrial oxidative phosphorylation and promoting ATP production in H_2_O_2_-injured astrocytes (Figure 5J).

## 4. Discussion

Extracellular mitochondria have been proposed to modulate cellular homeostasis after injury, but the mechanisms underlying their protective effects remain incompletely understood. In this study, we identify mitochondria-associated ribosomes and their bound mRNAs as a translation-dependent mechanism that supports ATP recovery during oxidative stress. By directly comparing unmodified mitochondria, MitoCoat, and MitoCoat–mRNA, we demonstrate that mitochondrial structural delivery and mRNA delivery represent mechanistically distinct modes of metabolic support.

Unmodified mitochondria failed to restore ATP under oxidative stress and instead elicited broad transcriptional responses associated with innate immune activation and cytoskeletal remodeling. These findings are consistent with prior observations that extracellular or compromised mitochondria engage cellular surveillance and stress pathways [24,25,26]. The induction of such transcriptional programs coincided with incomplete ATP recovery, suggesting that direct mitochondrial delivery may impose a substantial energetic burden on injured cells. In contrast, lipid modification fundamentally altered these outcomes. MitoCoat attenuated immune and stress-response signatures while enriching pathways related to ribosomal function and metabolic support. This more restricted transcriptional profile coincided with effective ATP restoration, suggesting that lipid coating redirects mitochondrial uptake and intracellular handling toward lower-burden routes that permit productive utilization of delivered material.

Beyond mitigating delivery-associated stress responses, our data reveal an autonomous contribution of mitochondria-associated mRNAs to metabolic recovery. MitoCoat–mRNA restored ATP levels without inducing detectable changes in the host transcriptome, indicating that nuclear gene reprogramming is not required for energy rescue under oxidative stress. Consistent with this interpretation, the enzymatic removal of mitochondrial surface-associated ribosomes and mRNAs significantly attenuated ATP restoration, and the pharmacological inhibition of cytosolic—but not mitochondrial—translation abolished mRNA-mediated rescue. Among the metabolic enzymes examined, PFKP, PDH, and ATP synthase subunit ATP5A showed the most consistent restoration following mitochondria-associated mRNA delivery. PFKP, a rate-limiting glycolytic enzyme, governs glucose commitment and upstream energy flux. Its restoration suggests that mRNA delivery enhances glycolytic throughput alongside mitochondrial function, increasing substrate supply to downstream pathways [27]. Because PDH controls the entry of glycolytic carbon into the TCA cycle and ATP5A catalyzes ATP synthesis downstream of OXPHOS, their coordinated recovery identifies key metabolic nodes through which mitochondria-associated mRNAs re-establish coupling between glycolysis and mitochondrial ATP production. Together, these findings identify cytosolic translation as the principal effector process underlying mRNA-driven ATP recovery.

These observations align with emerging models of localized translation at the mitochondrial surface, in which cytosolic ribosomes associate with the outer mitochondrial membrane to support the co-translational import of nuclear-encoded mitochondrial proteins via the TOM complex [11,28,29]. Our data extend this paradigm by demonstrating that exogenously delivered mitochondria-associated mRNAs can function as a functionally sufficient metabolic support module, as these transcripts were detected in close proximity to mitochondria and translated by cytosolic translation machinery in injured astrocytes. This mechanism also distinguishes component delivery from whole-mitochondria transplantation. Because the preparation and handling of intact mitochondria can be technically challenging in acute clinical settings [30], the delivery of intact mitochondria via MitoCoat may be better suited to supporting metabolic restoration during subacute or chronic phases of injury or disease. In contrast, because these transcripts can be stored until treatment, the delivery of mitochondria-associated mRNAs may enable rapid ATP restoration in acute injury or disease contexts.

Several limitations warrant consideration. First, although we identified three consistently restored proteins such as PFKP, PDH, and ATP5A following mitochondria-associated mRNA delivery, their causal contribution to ATP recovery was not directly tested. Future studies using targeted depletion or enrichment of candidate transcripts will be required to define causal contributions of individual mRNAs. Second, the relatively small number of biological replicates in the transcriptomic analyses (n = 3 per group) may limit the power to detect modest gene expression changes. Therefore, these findings should be considered hypothesis-generating and supported by pathway-level analyses and functional assays. Third, the precise intracellular sites of mRNA translation remain undefined, although the absence of host transcriptional changes and the dependence on cytosolic translation support a post-transcriptional mechanism. Finally, this study was conducted in an acute in vitro oxidative stress model, and the durability, biodistribution, and safety of these approaches in vivo remain to be determined. In addition, successful clinical translation will require organ- or cell-specific targeting. The MitoCoat platform [16] may offer flexibility in this regard, as its lipid components can be modified beyond DOTAP/DOPE and its surface engineered to enable targeted delivery, including antibody conjugation [31]. Collectively, while these findings highlight the therapeutic potential of mitochondria-associated mRNA delivery and the MitoCoat platform, further mechanistic, proteomic, and in vivo studies are needed to establish efficacy, specificity, and translational feasibility.

In summary, our findings identify mitochondria-associated mRNAs as translation-dependent mediators of metabolic support that operate independently of nuclear gene reprogramming. These results expand the conceptual framework of extracellular mitochondrial biology by demonstrating that mitochondria-associated mRNAs function as therapeutic metabolic effectors that preserve cellular energy homeostasis during oxidative stress.

## Data Availability

RNA-seq data have been deposited in the NCBI Gene Expression Omnibus under accession number GSE326220 and in the Sequence Read Archive under BioProject accession number PRJNA1443374.

## Supplementary Materials

The following supporting information can be downloaded at: https://www.mdpi.com/article/10.3390/antiox15050580/s1, Figure S1: MitoCoat and Mitochondria-associated mRNAs improve astrocytic viability after oxidative stress.
