# Supplementary material for: Mitochondria-Associated mRNAs Restore ATP During Oxidative Stress via Cytosolic Translation
Source: Antioxidants (Basel). 2026 May 3;15(5):580. doi: 10.3390/antiox15050580 (PMC13203415; doi:10.3390/antiox15050580)
Supplement: Supplementary file 1 [file antioxidants-15-00580-s001.zip › antioxidants-4248130-supplementary.pdf]

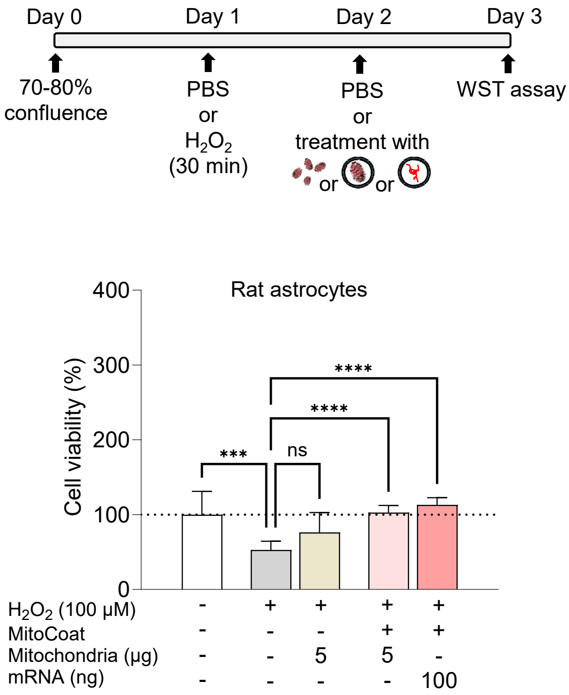

Figure S1: MitoCoat and Mitochondria-associated mRNAs improve astrocytic viability after oxidative stress.
